# Association between the triglyceride glucose index and cognitive impairment and dementia: a meta-analysis

**DOI:** 10.3389/fnagi.2023.1278730

**Published:** 2023-12-14

**Authors:** Huan Wang, Qin Ling, Yifan Wu, Mingjie Zhang

**Affiliations:** ^1^Department of Geriatrics, Liaoning Jinqiu Hospital, Shenyang, China; ^2^Second Clinical Medical College of Nanchang University, Nanchang, China; ^3^Department of Neurosurgery, Shengjing Hospital of China Medical University, Shenyang, China

**Keywords:** triglyceride and glucose index, cognitive impairment, dementia, insulin resistance mild cognitive impairment, insulin resistance triglyceride and glucose index, insulin resistance

## Abstract

**Background:**

The triglyceride and glucose (TyG) index is an alternative index of insulin resistance (IR). We aimed to clarify the relationship between the TyG index and cognitive impairment and dementia.

**Methods:**

We conducted a comprehensive search of the PubMed, Cochrane Library, and Embase databases until February 2023 to identify relevant studies. Random-effects models were used to pool effect sizes, and the Grading of Recommendations Assessment, Development, and Evaluation system (GRADE) was used to assess the quality of the evidence.

**Results:**

Ten studies were included, with seven of which investigated the relationship between the TyG index and cognitive impairment and three exploring the association between the TyG index and dementia. When the TyG index was described as a categorical variable, it was positively associated with the risk of cognitive impairment (OR = 2.32; 95% CI 1.39–3.87) and dementia (OR = 1.14, 95% CI 1.12–1.16). The association of the TyG index with the risk of cognitive impairment (OR = 3.39, 95% CI 1.67–6.84) and dementia (OR = 1.37, 95% CI 1.03–1.83) remained significant for per 1 unit increment in the TyG index. The GRADE assessment indicated a very low certainty for cognitive impairment. Low certainty and moderate certainty were observed for dementia when the TyG index was analyzed as a categorical variable and as a continuous variable, respectively.

**Conclusion:**

The TyG index is associated with an increased risk of cognitive impairment and dementia. Further prospective research is warranted to confirm these findings.

Systematic review registration: https://www.crd.york.ac.uk/, Protocol registration number: CRD42023388028.

## Introduction

As the global population ages, cognitive impairment has emerged as a pressing public health concern. Age-related dementia is projected to affect 150 million individuals by 2050 (Iadecola et al., 2019). Mild cognitive impairment (MCI) often precedes dementia, imposing substantial treatment costs and significantly diminishing patients’ quality of life. Therefore, identifying simple risk factor are imperative for the cognitive impairment and dementia screening and early diagnosis.

Insulin resistance (IR) has long been recognized as an important risk factor for the development of neurodegeneration and cognitive impairment (Cui et al., 2022). The triglyceride and glucose (TyG) index, as an applicable indicator of IR (Guerrero-Romero et al., 2010), is highly likely to be closely associated with cognitive impairment and dementia, as shown by many previous studies. For instance, Weyman-Vela et al. found that a high TyG index was strongly associated with MCI in older adults (Weyman-Vela et al., 2022). A prospective cohort study conducted by Sun et al. also proposed that the TyG index may be used as a simple surrogate marker for early detection of Alzheimer’s disease (AD; Sun et al., 2022). However, as far as we know, there is no existing meta-analysis to systematically assess the relationship between the TyG index and cognitive impairment and dementia. As a result, we aimed to conduct a systematic meta-analysis to evaluate the association between the TyG index and cognitive impairment and dementia.

## Methods

### Protocol and registration

We registered our protocol (registration number: CRD42023388028) at PROSPERO (International Prospective Register of Systematic Reviews)^1^ system. We reported our results by following the Preferred Reporting Items for Systematic Reviews and Meta-analysis (PRISMA) (Supplementary Table S1).

### Literature search

We conducted a careful search of all studies that reported the relationship between the TyG index and cognitive impairment and dementia in PubMed,^2^ Cochrane Library^3^ and Embase^4^ until the end of February 2023. The search terms were “Cognitive Dysfunction” OR “Cognitive Impairment” OR “Cognitive Disorder” OR “Mild Cognitive Impairment” OR “Cognitive Decline” OR “Mental Deterioration” OR “Dementia” AND “TyG index” OR “triglyceride glucose index” OR “triacylglycerol glucose index.” The detailed search strategy is presented in Supplementary Table S2.

### Study selection

Two of our authors (MJ. Z and Q.L.) independently conducted the literature search and imported all relevant literature into Endnote X9 software (Tomson Reuters, New York, NY, United States). Duplicated documents were automatically and manually removed. The titles and abstracts of all articles were first screened, and content-related studies were read in full text to identify the included studies. In cases where no article or other information was available, we contacted the corresponding author for the information. The final included studies were determined by consensus between the two authors or resolved by the third reviewer (X. L.) if differences remained after consultation.

The Inclusion criteria were as follows: (1) Types of participants: adult (age > 18 years); (2) exposure and comparator: high TyG index versus low TyG index; (3) Outcomes: cognitive impairment or dementia; (4) types of studies: retrospective or prospective cohort, case–control and cross-sectional studies; and (5) the studies provided odds ratio (OR)/relative risk (RR)/hazard ratio (HR) and corresponding 95% confidence interval (CI) for the association between TyG Index and cognitive impairment and dementia.

Studies that meet any of the following criteria were excluded: (1) protocols, reviews, conference abstracts or animal studies; (2) studies that lacked a clear definition for cognitive impairment or dementia; (3) studies that used regression coefficients, SE, β or other methods for the statistical analysis; (4) studies for which relevant data could not be obtained completely; and (5) studies with unavailable data even after contacting the corresponding author for further information.

### Data extraction and quality assessment

We extracted the following information from the included studies: (1) first author’s last name; (2) publication year; (3) country or region; (4) study design; (5) participants’ characteristics (source, mean age, sex, etc.); (6) outcome; (7) diagnosis; (8) categories of TyG; (9) hazard ratio (HR) or odds ratio (OR) from the most adjusted model (with 95% confidence interval (CI)); (10) follow-up period; and (11) adjustments.

The Newcastle Ottawa Quality Assessment Scale (NOS) was used to assess the quality of the included case–control and cohort studies, and studies with more than six stars were considered high-quality, with a total score of 9 stars. The included cross-sectional studies were assessed by Joanna Briggs Institute’s (JBI) critical appraisal checklist, which helps us to evaluate the quality by sampling method, size, diagnosis, measurement, and analysis. We assessed the quality and strength of evidence by the Grading of Recommendations Assessment, Development, and Evaluation (GRADE) (Atkins et al., 2004). GRADEPro GDT^5^ was used to provide evidence analysis tables.

### Statistical analysis

Most of the included studies reported the results with ORs, while a minority calculated HRs as the results. Therefore, we treated HRs as ORs because the incidence of cognitive or dementia is considered low (Shigesi et al., 2019) and finally used summary ORs and their corresponding 95% CIs as a general indicator of the association between the TyG index and cognitive impairment and dementia.

The formula of TyG: $\ln\left[\frac{\mathrm{triglycerides}\;\left(\mathrm{mg}/\mathrm{dL}\right)\times \mathrm{fasting}\;\mathrm{glucose}\;\left(\mathrm{mg}/\mathrm{dL}\right)}{2}\right]\text{.}$ For the studies in which the TyG index was described as a categorical variable, we compared the effect size of the highest TyG group to that of the lowest group, while we standardized the TyG index as per 1 unit in the studies that described the TyG index as a continuous variable. We summarized the effect size and conducted a subgroup analysis using RevMan software, version 5.4.1 (The Cochrane Collaboration 2016, Nordic Cochrane Center Copenhagen, Denmark) in a random-effects model, which followed Der Simonian and Laird’s generic inverse variance method (Xu et al., 2019).

We used *I*^2^ to explore the inconsistency across the findings of the included studies and Tau^2^ was reported as the variance of the true effect size. Since the number of studies we included for each outcome was less than 10, potential publication bias was not assessed.

Given the limited number of included studies, we only performed a subgroup analysis of the five studies that assessed the association between the TyG index and cognitive impairment, stratified by mean age, type of study design, sample size, body mass index (BMI), diagnosis and adjustment for confounders. Stata software (Version 16.0, Stata Corp LP, College Station, Texas, United States) was used to conduct sensitivity analyses.

## Results

### Literature search

**Figure 1:**
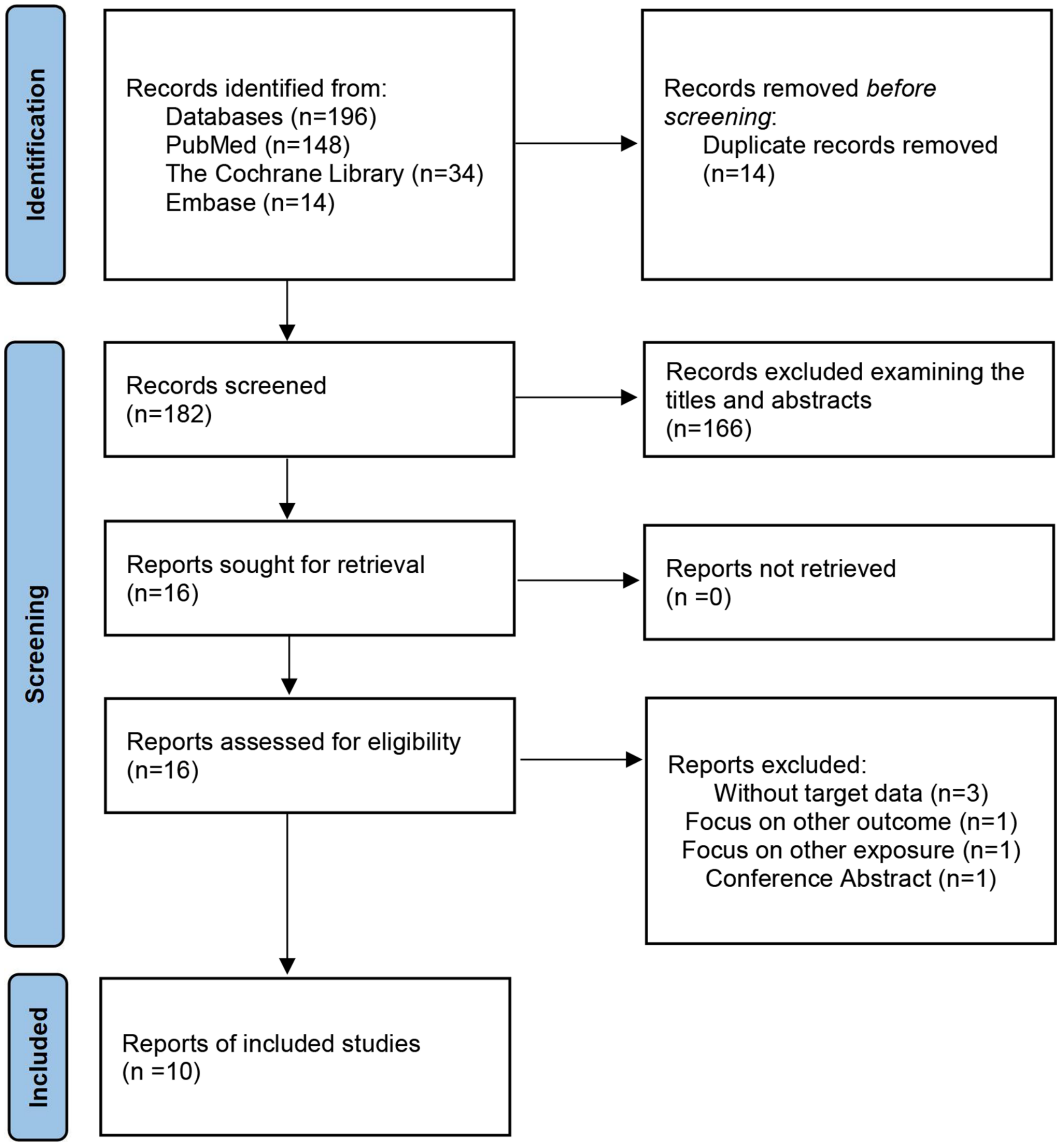
Flow chart of the study selection process for the meta-analysis for the association between TyG index and cognitive impairment and dementia.

We retrieved a total of 196 related studies in these three databases (PubMed = 148; Cochrane Library = 34; Embase = 14), and the overall inclusion flow of the studies is presented in Figure 1. After excluding 14 duplicate records, we screened the titles and abstracts of the remaining 182 articles, and ultimately, 16 articles were included for full-text review. Six of these articles were excluded for the following reasons: (1) they were without target data (*n* = 3); (2) it was focused on other outcomes (*n* = 1); (3) it was focused on other exposures (*n* = 1); and (4) it was a conference abstract (*n* = 1). The detailed reasons for exclusion for each of these studies are shown in Supplementary Table S3. We finally established the inclusion of seven cohort studies (Faqih et al., 2021; Guo et al., 2021; Hong et al., 2021; Li et al., 2022; Sun et al., 2022; Teng et al., 2022; Wang et al., 2022), one case–control study (Jiang et al., 2021) and two cross-sectional studies (Tong et al., 2022; Weyman-Vela et al., 2022).

### Study characteristics and quality evaluation

Table 1 summarizes the basic characteristics of the included studies according to their outcomes. The studies were published between 2021 and 2022 and included a total of 8,443,279 participants ranging in age from 58.2 to 80.5 years old. Only one study was conducted in North America (Weyman-Vela et al., 2022), while the remaining studies were conducted in Asia. Two of the included studies (Tong et al., 2022; Weyman-Vela et al., 2022) were cross-sectional studies, and the rest were cohort studies, which improved the credibility of our results. Among the studies, seven reported the association between the TyG index and cognitive impairment (Guo et al., 2021; Jiang et al., 2021; Li et al., 2022; Teng et al., 2022; Wang et al., 2022; Weyman-Vela et al., 2022), while the remaining four studies’ endpoints can be classified as “dementia” (Faqih et al., 2021; Hong et al., 2021; Sun et al., 2022; Tong et al., 2022). Cognitive impairment was mainly diagnosed by the Montreal Cognitive Assessment (MoCA) and Mini-Mental State Examination (MMSE), while the diagnosis of dementia was mostly based on the ICD-10. Each study adjusted for confounders that may influence the stability of the results.

**Table 1:** Characteristics of included studies in the meta-analysis of TyG and risk of cognitive impairment and dementia.

First Author, Year, Country	Source of participants	Participant	Study design/sample size	Mean age (years), Male (%)	Mean BMI, kg/m^2^	Outcome/diagnosis	Categories of TyG	OR/HR (95% CIs)	Follow-up period	Adjustments
**Cognitive impairment**										
Guo, 2021, China	Second Affiliated Hospital of Nantong University	Patients with CSVD	Retrospective cohort study/275	67.7, 54.5	24.5	VCI/MoCA: ≤26	<8.78 ≥8.78 Continuous variable	Ref 4.09 (2.18, 7.68) 2.42 (1.37, 4.29)	NR	Age, education level, LDL-C, homocysteine, SUA, and CSVD
Jiang, 2021, China	Second Affiliated Hospital of Nantong University	Patients with CSVD	Retrospective cohort study/280	67.6, 57.9	25	VCI/MoCA: ≤26	3.57 3.89 4.09 4.38	Ref 2.69 (1.17, 6.16) 2.54 (1.12, 5.75) 4.67 (1.79, 12.16)	NR	Age, sex, BMI, diabetes, hypertension, education level, HDL-C, LDL-C, Hs-CRP, HbAlc, IL-34 level, mRS, BI
Li, 2022, China	Jidong Cognitive Impairment Cohort Study	General population	Prospective cohort study/1,774	53.5, 48.0	25	Cognitive impairment/MMSE^a^	7.46 8.06 8.47 9.09	Ref 1.17 (0.85, 1.62) 1.31 (0.93, 1.83) 1.51 (1.06, 2.14)	4 years	Age, sex, BMI, educational level, smoking status, alcohol consumption, physical activity, history of hypertension, TG
Teng, 2022, China	Hebei General Hospital	Patients with T2D	Retrospective cohort study/308	71.0, 48.7	25	Cognitive impairment/MMSE^b^	18.39 28.96 39.58 Continuous variable	Ref 1.75 (0.93, 3.30) 3.30 (1.69, 6.45) 2.24 (1.44, 3.50)	5 years	Age, sex, education level, hypertension, history of stroke, SBP, HbA1c, HDL-C, insulin or metformin use, serum tHcy
Wang, 2022, China	China Health and Retirement Longitudinal Study	General population	Prospective cohort study/4,420	58.9, 46.7	NR	Cognitive impairment/WRT and MST: the slope of cognitive decline <0	Male: Q1 Q2 Q3 Q4 Female: Q1 Q2 Q3 Q4	Ref 1.13 (0.88, 1.45) 1.01 (0.79, 1.29) 1.11 (0.87, 1.42) Ref 1.09 (0.85, 1.40) 1.09 (0.85, 1.41) 1.32 (1.03, 1.71)	4 years	Age, sex, BMI, education level, marriage, residence, leisure time social activity, health insurance status, alcohol consumption, smoking status, hypertension, diabetes
Weyman-Vela, 2022, Mexico	Inhabitants from durango	General population	Cross-sectional study/135	72.8, 18.5	28.7	MCI/MMSE: 20–30	Continuous variable	2.97 (1.12, 14.71)	NA	Age, sex, WC, education level, occupation, physical activity
Tong, 2022, China	The First Affiliated Hospital of Harbin Medical University	Patients with T2D	Cross-sectional study/517	58.0, 54.4	25.3	MCI/National Institute on Aging-Alzheimer’s Association criteria	Continuous variable	7.37 (4.72, 11.50)	NA	Age, sex, smoking status, drinking consumption, duration of diabetes, education level, TG, HbA1c, diabetic nephropathy, fatty liver, insulin or statins use
**Dementia**										
Hong, 2021, Korea	National Health Screening program	General population	Retrospective cohort study/ 8,433,046	45.0, 55.7	23.5	All-cause dementia/ICD-10 for dementia	Male: 7.92 8.51 8.91 9.52 Female: 7.68 8.23 8.6 9.16	Ref 1.04 (1.02, 1.05) 1.07 (1.05, 1.09) 1.14 (1.12, 1.16) Ref 1.04 (1.02, 1.05) 1.07 (1.05, 1.09) 1.14 (1.12, 1.16)	7.21 years	Age, sex, BMI, smoking status, alcohol consumption, physical activity, low income, hypertension, TG
Sun, 2022, China	Framingham Heart Study Offspring cohort	General population	Prospective cohort study/2,170	63.0, 46.7	28.1	AD/NINCDS-ADRDA	7.62 8.48 8.89 10.55 Continuous variable	Ref 1.52 (0.93, 2.48) 1.69 (1.03, 2.77) 1.48 (0.86, 2.54) 1.39 (1.02, 1.88)	13.8 years	Age, sex, BMI, education level, smoking status, physical activity, SBP, CVD, antihypertensives, hypoglycemic therapy, lipid-lowering therapy
Faqih, 2021, Saudi Arabia	KAMC-J	General population	Retrospective cohort study/354	80.5, 46.3	NR	AD/ICD-10 for dementia	Continuous variable	1.2 (0.99, 3.1)	4 months	Age, sex, BMI, co-morbidities, insulin use, HbA1c

BMI, body mass index; TyG, triglyceride and glucose index; OR, odds ratio; HR, hazards ratio; CI, confidence interval; NA, not appliable; NR, not reported; AD, Alzheimer’s disease; LDL-C, low density lipoprotein cholesterol; SUA, serum uric acid; CVSD, cerebral small-vessel disease; VCI, vascular cognitive impairment; MoCA, Montreal Cognitive Assessment; TG, total cholesterol; HDL-C, high density lipoprotein cholesterol; Hs-CRP, high-sensitivity C-reactive protein; HbA1c, hemoglobin A1c; mRS, modified Rankin Scale; BI, Barthel Index; MMSE, Mini-Mental State Examination; SBP, systolic blood pressure; WRT, word recall test; MST, mental status test; CVD, cardiovascular disease; NINCDS-ADRDA, National Institute of Neurological and Communicative Disorders and Stroke and the AD and Related Disorders Association; T2D, type 2 diabetes; MCI, mild cognitive impairment; WC, waist circumference; KAMC-J, King Abdulaziz Medical City, Jeddah.^a^: ≤17 for illiterate individuals, ≤20 for primary school graduates, ≤24 for junior high school graduates or above.^b^: ≤17 for illiterate, ≤20 for patients with1-6 years of education, ≤24 for patients with 7 or more years of education.

**Figure 2:**
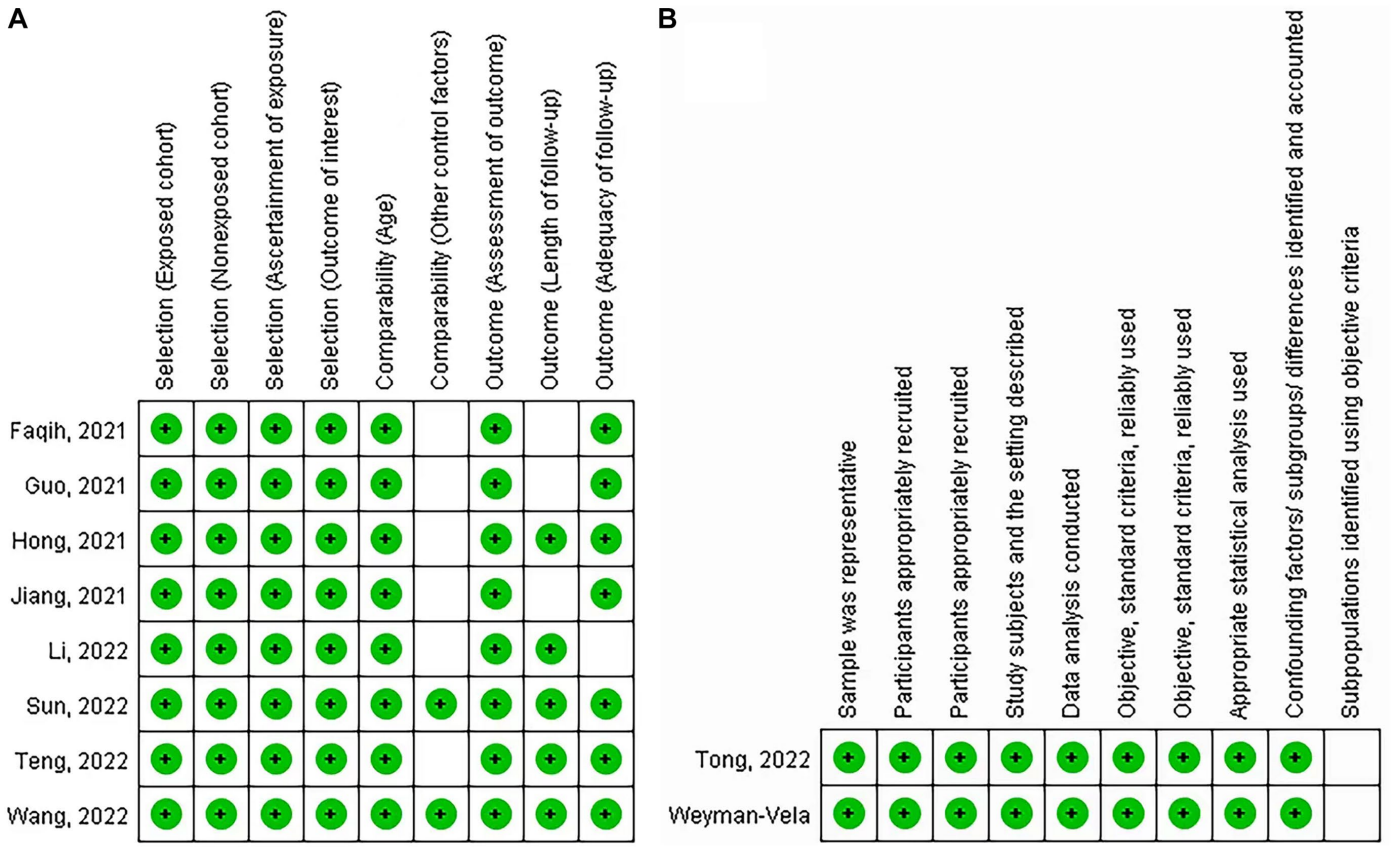
**(A)** The quality asessment of the included studies. Newcastle Ottawa Quality Assessment Scale for the case–control and cohort studies and **(B)** Joanna Briggs Institute’s critical appraisal checklist for cross-sectional studies. Each green pattern represents a score.

The JBI checklist for quality assessment of the two cross-sectional included studies is presented in Figure 2A, which shows that the quality was high in terms of sample representativeness, sample size, method, statistical analysis, and confounding factors. However, the criteria for the subgroup analysis were unclear. The NOS scores ranged from 7 to 9 (Figure 2B), suggesting that the quality of our included studies was acceptable.

### Association between the TyG index and cognitive impairment

**Figure 3:**
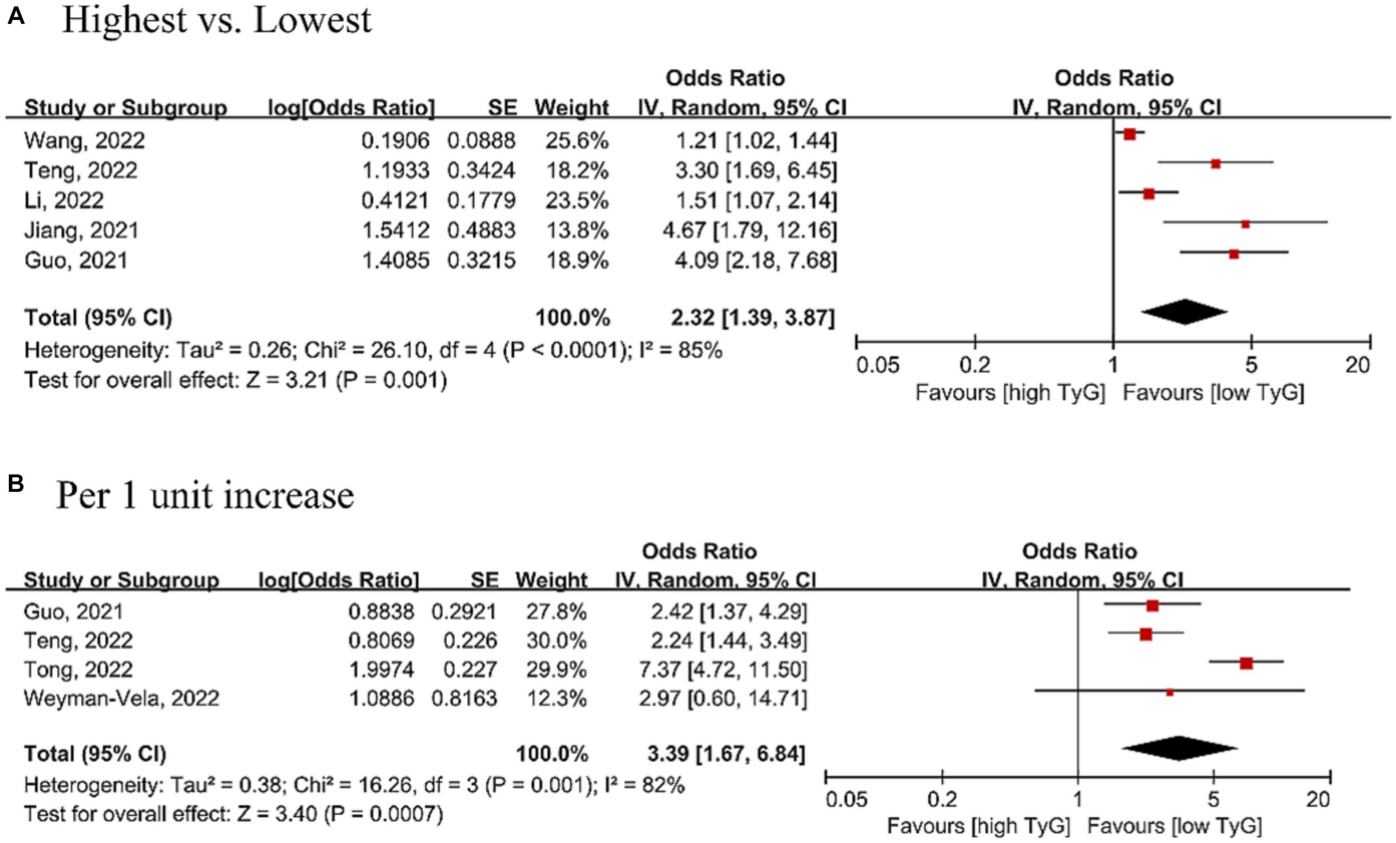
Forest plot of the association between the triglyceride-glucose index [**(A)** Analyzed as categorical variable; **(B)** analyzed as continuous variable] and the risk of cognitive impairment. The black midline indicates the line of no effect. The diamond indicates the pooled estimate. Red boxes are relative to study size, and the black transverse lines indicate the 95% confidence interval around the effect size estimate.

Seven studies with 7,709 participants were included in the analysis between the TyG index and cognitive impairment. The summary OR was 2.32 (95% CI 1.39–3.87, *p* < 0.001, τ^2^ = 0.26, highest vs. lowest), suggesting that a high TyG index was associated with a 132% higher risk of cognitive impairment (Figure 3A). Similarly, when the TyG index was analyzed as a continuous variable, we found that every unit of increasing TyG index may be associated with a 239% higher risk of cognitive impairment (OR = 3.39, 95% CI 1.67–6.84, *p* = 0.001, τ^2^ = 0.38; Figure 3B).

### Association between the TyG index and dementia

**Figure 4:**
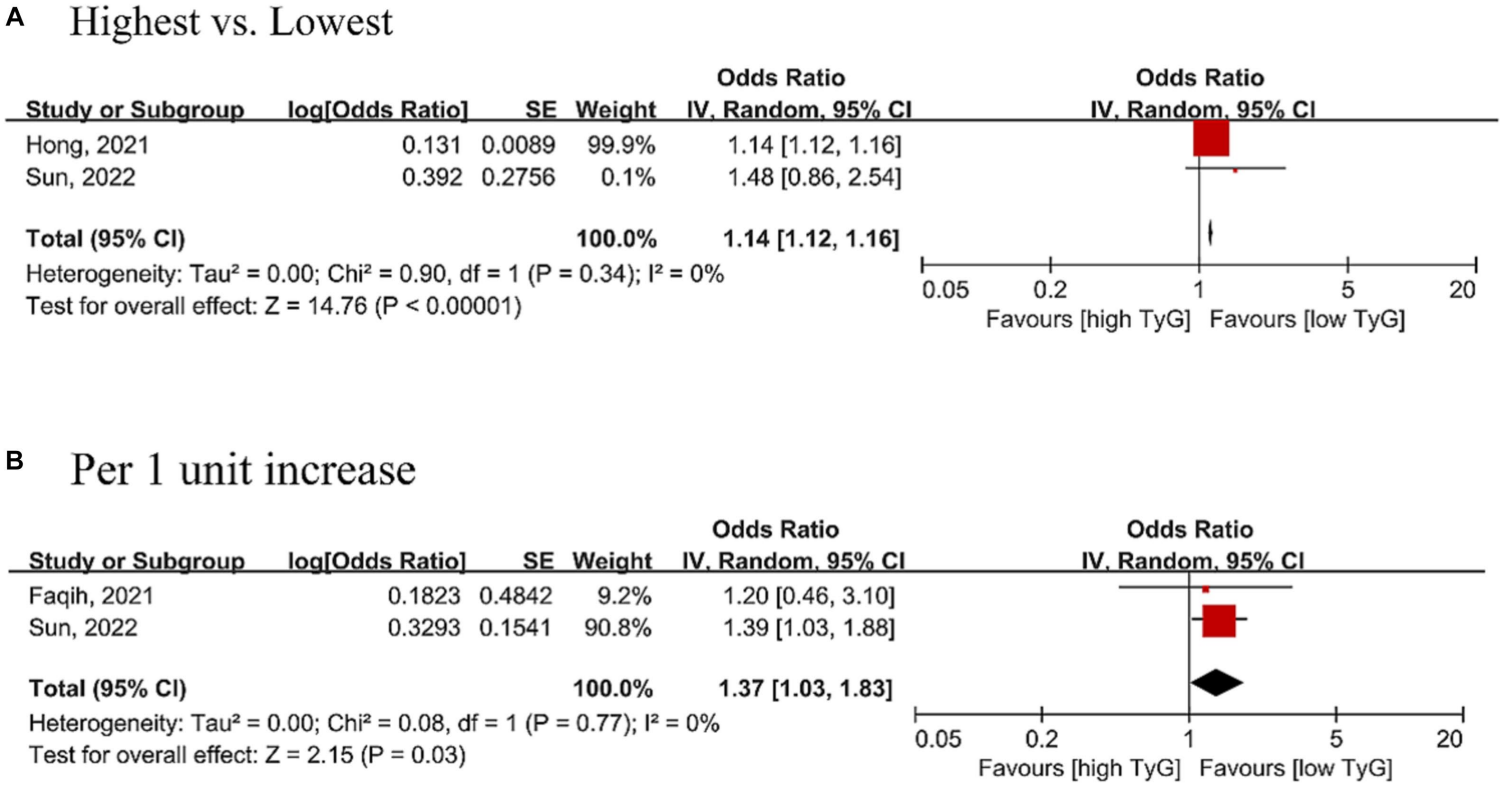
Forest plot of the association between the triglyceride-glucose index [**(A)** Analyzed as categorical variable; **(B)** analyzed as continuous variable] and the risk of dementia. The black midline indicates the line of no effect. The diamond indicates the pooled estimate. Red boxes are relative to study size, and the black transverse lines indicate the 95% confidence interval around the effect size estimate.

Three cohort studies with 8,435,570 participants were included for the association between the TyG index and dementia. TyG may be associated with the risk of dementia (OR = 1.14, 95% CI 1.12–1.16, *p* = 0.34, τ^2^ = 0) in the categorical analysis (Figure 4A). In studies analyzing the TyG index as a continuous variable, a 1.37 times higher risk of dementia may be associated with each increased unit of the TyG index, with OR = 1.37 (95% CI 1.03–1.83, *p* = 0.77, τ^2^ = 0) (Figure 4B).

### Sensitivity analysis and publication bias

A sensitivity analysis was performed by omitting one study at a time and revealed that the pooled results remained consistent in the association between the TyG index and cognitive impairment or dementia (Supplementary Figure S1), further supporting the validity of our main results. As we included a limited number of studies (fewer than 10 for each outcome), an analysis of publication bias was not performed.

### Subgroup analyses

Heterogeneity was not evident among the age-stratified subgroups, and similar results were shown in the sample-size-stratified groups and whether the model was adjusted for cholesterol (Table 2), suggesting that these three factors may be potential sources of heterogeneity.

**Table 2:** Subgroup analysis of TyG index and cognitive impairment.

Items		Number of studies	Effect size (95%CI)	*p* ^*^_h (%)_
Result of primary analysis		5	2.32 [1.39, 3.87]	85
Mean age	<60 years	2	1.28 [1.06, 1.55]	19
	≥60 years	3	3.86 [2.55, 5.84]	0
Sample size	<1,000	3	3.86 [2.55, 5.84]	0
	≥1,000	2	1.28 [1.06, 1.55]	19
BMI	<25	1	4.09 [2.18, 7.68]	–
	≥25	3	2.60 [1.27, 5.30]	74
	NR	1	1.21 [1.02, 1.44]	-
Diagnosis	MoCA	2	4.26 [2.52, 7.21]	0
	MMSE	2	2.11 [0.99, 4.51]	76
	Others	1	1.21 [1.02, 1.44]	–
*Adjustment for confounders*				
Gender	Yes	4	1.95 [1.20, 3.17]	80
	No	1	4.09 [2.18, 7.68]	–
Cerebrovascular disease	Yes	2	3.70 [2.34, 5.85]	0
	No	3	1.62 [1.03, 2.56]	76
Hypertension	Yes	4	1.95 [1.20, 3.17]	80
	No	1	4.09 [2.18, 7.68]	–
Diabetes	Yes	2	2.18 [0.59, 8.11]	86
	No	3	2.61 [1.32, 5.17]	79
Cholesterol	Yes	3	3.86 [2.55, 5.84]	0
	No	2	1.28 [1.06, 1.55]	19
Medication use	Yes	1	3.30 [1.69, 6.45]	-
	No	4	2.13 [1.23, 3.67]	85
Physical activity	Yes	1	1.51 [1.07, 2.14]	-
	No	4	2.79 [1.24, 6.27]	88

*p for within-group heterogeneity (TyG index was analyzed as a categorical variable). TyG, triglyceride and glucose index; CI, confidence interval; BMI, body mass index; SBP, systolic blood pressure; NR, not report.

### GRADE assessment

The GRADE assessment revealed a very low level of certainty regarding the association between the TyG index and cognitive impairment due to significant heterogeneity observed in both the categorical analysis (*p* < 0.001) and the continuous analysis (*p* = 0.001). Moreover, the lack of serious risk of bias warranted a downgrade. On the other hand, the GRADE assessment indicated a moderate level of certainty regarding the association between the TyG index and dementia when analyzed as a continuous variable (Supplementary Table S4).

## Discussion

### Major findings

Our study found that a higher TyG index was significantly associated with an increased risk of cognitive impairment and dementia. When the TyG index was regarded as a categorical variable, the risk of cognitive impairment and dementia in the high TyG index group was 2.32 and 1.14 times higher than that in the low TyG index group, respectively. For each additional unit of the TyG index, the risk for cognitive impairment and dementia increased by 3.39 and 1.37, respectively. To the best of our knowledge, this is the first meta-analysis of the association between the TyG index and cognitive impairment and dementia.

To account for the potential impact of various factors on cognitive impairment, we conducted a subgroup analysis. Currently, cognitive impairment is typically diagnosed using assessment scales such as the MMSE and the MoCA. The use of different scales across studies could potentially affect our results. However, our analysis showed a high level of within-group heterogeneity, particularly in the subgroup using the MMSE (76%), indicating that the diagnostic method for cognitive impairment is not the primary source of heterogeneity in our results. Nonetheless, given the limited number of studies included, it is worth further exploring whether diagnostic methods may impact the relationship between the TyG index and cognitive impairment.

In groups with a mean age older than 60 years, a sample size of less than 1,000, and adjusted cholesterol levels, there was more than a threefold greater risk of cognitive impairment associated with a higher TyG index. This is consistent with the higher incidence of cognitive impairment in elderly individuals (Pais et al., 2020). The within-group heterogeneity in these subgroups was small, suggesting that age and adjustment for high cholesterol were the primary sources of heterogeneity in our final results.

Moreover, a cohort study that enrolled 1,674 individuals showed that people who used statins were approximately 50% less likely to develop cognitive impairment or dementia than those who did not use statins (HR = 0.52; 95% CI 0.34–0.80; Cramer et al., 2008). Another study, the Sydney Memory and Aging Study (Samaras et al., 2020), found that the incidence of dementia was significantly higher in diabetes patients who did not receive metformin than in those who did (OR = 5.29, 95% CI 1.17–23.88). These findings suggest that hypoglycemic and hypolipidemic drugs may decrease the risk of cognitive impairment and dementia as well as affect the measurement of the TyG index. Therefore, we acknowledge that these factors may affect our results, and they should be considered in future studies. However, only a few included studies stated that they have adjusted for these factors. Due to the limited number of studies included in our analysis, we were unable to perform a subgroup analysis of these factors to explore whether they might affect our final results, so further investigation is necessary.

### Potential mechanism

Among the underlying mechanisms associated with the TyG index and cognitive impairment, IR may be one of the reasons that cause cognitive impairment independently or in combination with other pathologies.

First of all, IR may contribute to cognitive impairment uniquely. Prolonged IR can hinder insulin from crossing the blood–brain barrier (Arnold et al., 2018), thereby reducing insulin levels in the brain and then reducing the levels of insulin-degrading enzymes that break down amyloid-β (Aβ). As a hallmark of AD, Aβ is known to form insoluble extracellular plaques (Cole and Frautschy, 2007). So low insulin level can lead to its accumulation and potentially impair cognitive function (Ghasemi et al., 2014). In addition, low insulin levels or insulin insensitivity can result in the long-term inhibition (LTD) of excitatory synaptic transmission by regulating the endocytosis of 3-hydroxy-5-methylisoxazole-4-propionic acid (AMPA) receptors (Huang et al., 2010), which can reduce hippocampal synaptic plasticity, leading to memory loss or, even worse, neurodegenerative diseases.

Secondly, IR may also combine with other pathologies to promote the incidence of cognitive impairment. Previous studies have shown that patients with type II diabetes (T2D) have a higher risk of AD (Biessels et al., 2006; Luchsinger et al., 2007; Kopf and Frölich, 2009), which is characterized by IR, indicating that there may be a common pathological mechanism between T2D and AD. They are both protein misfolding disorders associated with protein aggregate deposition due to misfolding of protein structures (Chiti and Dobson, 2006), with Aβ depositing in the brain of AD patients and pancreatic amyloid peptides (IAPP) in the pancreas of T2D patients. A study hypothesized that IAPP and Aβ can interact through ‘cross-seeding’ to enhance the deposition of misfolded protein aggregates (Moreno-Gonzalez et al., 2017), which may explain the high incidence of comorbidities in AD and T2D.

Several studies have notably linked IR to reduced brain perfusion (Cui et al., 2017; Hoscheidt et al., 2017). In a small case–control study, individuals with type 2 diabetes exhibited significantly lower cerebral blood flow (CBF) compared to healthy controls. Additionally, a moderate correlation emerged between IR, as assessed by the HOMA-IR index, and decreased perfusion in posterior brain regions, particularly the posterior cingulate cortex and precuneus, as revealed by MRI. Since hypoperfusion is known to be associated with the development and advancement of neuropathologies, IR may contribute to cognitive impairment through its involvement in hypoperfusion.

### Clinical implications

Our study found a link between an elevated TyG index and the risk of cognitive impairment and dementia. The TyG index values can be easily obtained in clinical practice by measuring plasma glucose and TG levels. Several studies have suggested that the TyG index has the potential to serve as an indicator of cognitive impairment.

In a study by Jiang et al. (2021), the diagnostic performance of the TyG index for vascular cognitive impairment (VCI) was evaluated using ROC curve analysis. The optimal threshold was determined to be 3.94, with an area under the curve (AUC) of 0.727 (95% CI 0.636–0.779). Similarly, Teng et al. (2022) identified an optimal cutoff point of 9.015 for the TyG index in diagnosing cognitive impairment, with an AUC of 0.671. However, it is important to note that these studies were primarily preclinical and had limitations such as small sample sizes, limited generalizability to different races and regions, and study design. Therefore, further research is needed to fully understand the predictive value of the TyG index in dementia or Alzheimer’s disease.

## Limitations

We acknowledge that there were some limitations in our study. First, the number of included studies was limited, future studies are needed to further validate our findings. Second, the majority of included studies were conducted in Asia, with only one study from Mexico. Therefore, the influence of geographical and racial factors on our results cannot be fully excluded, and further studies from diverse populations are needed to validate our results. Third, potential confounding variables such as sex, age, diabetes, hypertension, genetic factors, and others may affect our results. For example, the apolipoprotein E (APOE) ε4 allele is one of the most significant risk factors for cognitive impairment, accounting for about 5% of the variance in lifetime cognitive decline (Davies et al., 2014). Meanwhile, ApoE is involved in lipid transport and lipoprotein metabolism to mediate lipid distribution or redistribution in tissues and cells, affecting the plasma triglyceride level and then the TyG index level (Getz and Reardon, 2018). Thus, if the APOE genotypes in each of the included articles can be analyzed, it could be better to understand the relationship between TyG and cognitive impairment or dementia. Moreover, the TyG index was calculated from TG and plasma glucose, hypoglycemic and hypolipidemic drugs will affect the measurement of the TyG index. However, due to the data limitations, we cannot exclude the potential effect. Fourth, some of the included studies were cross-sectional in design. However, only two of the included studies (Tong et al., 2022; Weyman-Vela et al., 2022) were cross-sectional studies, and excluding them did not affect the final results (Supplementary Figure S1B). Finally, the data restriction precludes us from conducting an exposure-response analysis. More importantly, despite there are thousands of publicly published papers reporting the relationship between AD and T2D, observations from clinical studies are highly controversial and failed to elucidate a clear pathology pathway for an increased risk of dementia in patients with T2D (Salas and De Strooper, 2018). As a result, we need to pay more attention to the studies with different results and make rational analyses, to facilitate broader scientific consensus.

## Conclusion

In conclusion, our study showed that an elevated TyG index is associated with the risk of cognitive impairment and dementia. However, given the limitations of the included articles, additional studies are needed to explore the generality of the association of TyG with cognitive impairment and dementia. The GRADE assessment showed very low certainty for cognitive impairment, and low (categorical variable) or moderate (continuous variable) certainty for dementia with TyG index. Despite some limitations, our findings provide important insights into the association between the TyG index and cognitive impairment and dementia and highlight the need for further research in this area.

## Data availability statement

The original contributions presented in the study are included in the article/Supplementary material, further inquiries can be directed to the corresponding author.

## Author contributions

HW: Writing – original draft. QL: Writing – original draft. YW: Writing – original draft. MZ: Writing – review & editing.

## Glossary

**Table:** 

MCI	Mild cognitive impairment
IR	Insulin resistance
TyG	Triglyceride and glucose
AD	Alzheimer’s disease
OR	Odd ratio
RR	Relative risk
HR	Hazard ratio
CI	Confidence interval
NOS	Newcastle Ottawa Quality Assessment Scale
JBI	Joanna Briggs Institute’s
GRADE	Grading of Recommendations Assessment, Development, and Evaluation
BMI	Body mass index
MoCA	Montreal Cognitive Assessment
MMSE	Mini-Mental State Examination
Aβ	Amyloid-β
LTD	Long-term inhibition
T2D	Type II diabetes
CBF	Cerebral blood flow
VCI	Vascular cognitive impairment
AUC	Area under the curve
APOE	Apolipoprotein E

## Footnotes

^1^ http://www.york.ac.uk/ast/crd

^2^ https://pubmed.ncbi.nlm.nih.gov/

^3^ http://www.cochranelibrary.com

^4^ https://www.embase.com/

^5^ https://gradepro.org
