# Supplementary material for: Association between the triglyceride glucose index and cognitive impairment and dementia: a meta-analysis
Source: Front Aging Neurosci. 2023 Dec 14;15:1278730. doi: 10.3389/fnagi.2023.1278730 (PMC10757637; doi:10.3389/fnagi.2023.1278730)
Supplement: Supplementary file 1 [file Data_Sheet_1.docx]

**Data Supplement**

**Association between triglyceride glucose index (TyG index) and cognitive impairment and dementia: A meta-analysis**

Huan Wang*, M.D., Qin Ling*, M.D., Yifan Wu, M.D., Mingjie Zhang^5^, M.D.

Author Affiliations：

Department of Geriatrics, Liaoning Jinqiu Hospital, Shenyang,China (H.W.); Second Clinical Medical Colleage of Nanchang University, Nanchang, China (Q.L., Y.F.W.); Department of Neurosurgery, Shengjing Hospital of China Medical University, Shenyang, China (M.J.Z.)

* These authors contributed equally to this work as co-first authors.

^#^ These authors are senior authors.

Correspondence to Prof Mingjie Zhang, Department of Neurosurgery, Shengjing Hospital of China Medical University, Shenyang, China; Email:[mingjiezhang202304@163.com](mailto:mingjiezhang202304@163.com)

**Supplementary Table 1. PRISMA Checklist.**

| **Section/topic** | **#** | **Checklist item** | **Reported on page #** |
| --- | --- | --- | --- |
| **TITLE** | | |  |
| Title | 1 | Identify the report as a systematic review, meta-analysis, or both. | 1 |
| **ABSTRACT** | | |  |
| Structured summary | 2 | Provide a structured summary including, as applicable: background; objectives; data sources; study eligibility criteria, participants, and interventions; study appraisal and synthesis methods; results; limitations; conclusions and implications of key findings; systematic review registration number. | 3 |
| **INTRODUCTION** | | |  |
| Rationale | 3 | Describe the rationale for the review in the context of what is already known. | 5 |
| Objectives | 4 | Provide an explicit statement of questions being addressed with reference to participants, interventions, comparisons, outcomes, and study design (PICOS). | 5 |
| **METHODS** | | |  |
| Protocol and registration | 5 | Indicate if a review protocol exists, if and where it can be accessed (e.g., Web address), and, if available, provide registration information including registration number. | 6 |
| Eligibility criteria | 6 | Specify study characteristics (e.g., PICOS, length of follow-up) and report characteristics (e.g., years considered, language, publication status) used as criteria for eligibility, giving rationale. | 7 |
| Information sources | 7 | Describe all information sources (e.g., databases with dates of coverage, contact with study authors to identify additional studies) in the search and date last searched. | 6 |
| Search | 8 | Present full electronic search strategy for at least one database, including any limits used, such that it could be repeated. | 6 |
| Study selection | 9 | State the process for selecting studies (i.e., screening, eligibility, included in systematic review, and, if applicable, included in the meta-analysis). | 7 |
| Data collection process | 10 | Describe method of data extraction from reports (e.g., piloted forms, independently, in duplicate) and any processes for obtaining and confirming data from investigators. | 8 |
| Data items | 11 | List and define all variables for which data were sought (e.g., PICOS, funding sources) and any assumptions and simplifications made. | 8 |
| Risk of bias in individual studies | 12 | Describe methods used for assessing risk of bias of individual studies (including specification of whether this was done at the study or outcome level), and how this information is to be used in any data synthesis. | 9 |
| Summary measures | 13 | State the principal summary measures (e.g., risk ratio, difference in means). | 9 |
| Synthesis of results | 14 | Describe the methods of handling data and combining results of studies, if done, including measures of consistency (e.g., I^2^) for each meta-analysis. | 9 |
| Risk of bias across studies | 15 | Specify any assessment of risk of bias that may affect the cumulative evidence (e.g., publication bias, selective reporting within studies). | 9 |
| Additional analyses | 16 | Describe methods of additional analyses (e.g., sensitivity or subgroup analyses, meta-regression), if done, indicating which were pre-specified. | 9 |
| **RESULTS** | | |  |
| Study selection | 17 | Give numbers of studies screened, assessed for eligibility, and included in the review, with reasons for exclusions at each stage, ideally with a flow diagram. | 10 |
| Study characteristics | 18 | For each study, present characteristics for which data were extracted (e.g., study size, PICOS, follow-up period) and provide the citations. | 10 |
| Risk of bias within studies | 19 | Present data on risk of bias of each study and, if available, any outcome level assessment (see item 12). | 11 |
| Results of individual studies | 20 | For all outcomes considered (benefits or harms), present, for each study: (a) simple summary data for each intervention group (b) effect estimates and confidence intervals, ideally with a forest plot. | 11-12 |
| Synthesis of results | 21 | Present results of each meta-analysis done, including confidence intervals and measures of consistency. | 12 |
| Risk of bias across studies | 22 | Present results of any assessment of risk of bias across studies (see Item 15). | 12 |
| Additional analysis | 23 | Give results of additional analyses, if done (e.g., sensitivity or subgroup analyses, meta-regression [see Item 16]). | 12 |
| **DISCUSSION** | | |  |
| Summary of evidence | 24 | Summarize the main findings including the strength of evidence for each main outcome; consider their relevance to key groups (e.g., healthcare providers, users, and policy makers). | 13-16 |
| Limitations | 25 | Discuss limitations at study and outcome level (e.g., risk of bias), and at review-level (e.g., incomplete retrieval of identified research, reporting bias). | 17-18 |
| Conclusions | 26 | Provide a general interpretation of the results in the context of other evidence, and implications for future research. | 18 |
| **FUNDING** | | |  |
| Funding | 27 | Describe sources of funding for the systematic review and other support (e.g., supply of data); role of funders for the systematic review. | 19 |

*From:*  Moher D, Liberati A, Tetzlaff J, Altman DG, The PRISMA Group (2009). Preferred Reporting Items for Systematic Reviews and Meta-Analyses: The PRISMA Statement. PLoS Med 6(7): e1000097. doi:10.1371/journal.pmed1000097

For more information, visit: **www.prisma-statement.org**.

**Supplementary Table 2. Search strategy**

| Datebase | Search | Query |
| --- | --- | --- |
| PubMed database | #1 | ((((((Cognitive Dysfunction) OR (Cognitive Impairment)) OR (Cognitive Disorder)) OR (Mild Cognitive Impairment)) OR (Cognitive Decline)) OR (Mental Deterioration)) OR (Dementia) |
|  | #2 | (((TyG index) OR (triglyceride glucose index)) OR (triacylglycerol glucose index)) |
|  | #3 | #1 AND #2 |
| Embase database | #1 | 'cognitive defect'/exp |
|  | #2 | 'cognition disorder'/exp OR 'cognition disorders'/exp OR 'cognitive defects'/exp OR 'cognitive deficit'/exp OR 'cognitive disability'/exp OR 'cognitive disorder'/exp OR 'cognitive disorders'/exp OR 'cognitive dysfunction'/exp OR 'cognitive impairment'/exp OR 'delirium, dementia, amnestic, cognitive disorders'/exp OR 'overinclusion'/exp OR 'response interference'/exp |
|  | #3 | 'triglyceride-glucose index'/exp |
|  | #4 | 'fasting glucose and triglyceride (tyg index)' OR 'fasting plasma glucose and triglycerides (tyg index)' OR 'glucose and triglycerides (tyg index)' OR 'triglyceride and glucose (tyg) index' OR 'triglyceride and glucose index' |
|  | #5 | #1 OR #2 |
|  | #6 | #3 OR #4 |
|  | #7 | #5 AND #6 |
| Cochrane library | #1 | MeSH descriptor: [Cognitive Dysfunction] explode all trees |
|  | #2 | (Cognitive Dysfunction):ti,ab,kw |
|  | #3 | MeSH descriptor: [Dementia] explode all trees |
|  | #4 | (Dementia):ti,ab,kw |
|  | #5 | (triglyceride and glucose index): ti,ab,kw |
|  | #6 | #1 OR #2 OR #3 OR #4 |
|  | #7 | #5 AND #6 |

**Supplementary Table 3. Studies excluded (n=6) with reasons**

| **Studies excluded** | **Reasons (according to PICOS)** |
| --- | --- |
| Zhang, 2022[1] | Focus on other outcome: Cerebral small vessel disease |
| Huang, 2022[2] | Without target data set: Not providing OR, HR or RR |
| Gentreau, 2022[3] | Without target data set: Not providing OR, HR or RR |
| Chen,2022[4] | Without target data set: Not providing OR, HR or RR |
| Feinkohl, 2019[5] | Focus on other exposure: metabolic syndrome |
| Hong, 2019[6] | Conference Abstract |

[1] J. Zhang, M. Hu, Y. Jia, S. Zhao, P. Lv, M. Fan, Y. Shi, and W. Jin, The triglyceride glucose index is associated with the cerebral small vessel disease in a memory clinic population. J Clin Neurosci 104 (2022) 126-133.

[2] S.H. Huang, S.C. Chen, J.H. Geng, D.W. Wu, and C.H. Li, Metabolic Syndrome and High-Obesity-Related Indices Are Associated with Poor Cognitive Function in a Large Taiwanese Population Study Older than 60 Years. Nutrients 14 (2022).

[3] M. Gentreau, C. Reynes, R. Sabatier, J.J. Maller, C. Meslin, J. Deverdun, E. Le Bars, M. Raymond, C. Berticat, and S. Artero, Glucometabolic Changes Are Associated with Structural Gray Matter Alterations in Prodromal Dementia. Journal of Alzheimer's Disease 89 (2022) 1293-1302.

[4] C. Chen, Z. Lu, X. Wang, J. Zhang, D. Zhang, and S. Li, The chain mediating role of C-reactive protein and triglyceride-glucose index between lung function and cognitive function in a systemic low-grade inflammation state. Journal of psychiatric research 155 (2022) 380-386.

[5] I. Feinkohl, J. Janke, D. Hadzidiakos, A. Slooter, G. Winterer, C. Spies, and T. Pischon, Associations of the metabolic syndrome and its components with cognitive impairment in older adults. BMC geriatrics 19 (2019) 77.

[6] S.M. Hong, K. Han, and C.Y. Park, The triglyceride glucose index (TyG index), surrogate marker of insulin resistance and dementia: Population-based study. Diabetes 68 (2019).

**. Supplementary Table 4. GRADE evidence profile for the cardiovascular diseases and mortality.**

| **Certainty assessment** | | | | | | | **№ of patients** | | **Effect** | | **Certainty** | **Importance** |
| --- | --- | --- | --- | --- | --- | --- | --- | --- | --- | --- | --- | --- |
| **№ of studies** | **Study design** | **Risk of bias** | **Inconsistency** | **Indirectness** | **Imprecision** | **Other considerations** | **Outcome** | **Control** | **Relative**  **(95% CI)** | **Absolute**  **(95% CI)** |  |  |
| **association between the cognitive impairment and TyG index (analyzed as a categorical variable)** | | | | | | | | | | | | |
| 5 | observational studies | serious^a^ | serious^b^ | not serious | not serious | all plausible residual confounding would reduce the demonstrated effect |  |  | **OR 2.32**  (1.39 to 3.87) | **0 fewer per 1,000**  (from 0 fewer to 0 fewer) | ⊕OOO  Very low | CRITICAL |
| **association between the cognitive impairment and TyG index (analyzed as a continuous variable)** | | | | | | | | | | | | |
| 4 | observational studies | serious^a^ | serious^c^ | not serious | not serious | all plausible residual confounding would reduce the demonstrated effect |  |  | **OR 3.39**  (1.67 to 6.84) | **0 fewer per 1,000**  (from 0 fewer to 0 fewer) | ⊕OOO  Very low | CRITICAL |
| **association between the dementia and TyG index (analyzed as a categorical variable)** | | | | | | | | | | | | |
| 2 | observational studies | serious^a^ | not serious | not serious | not serious | all plausible residual confounding would reduce the demonstrated effect |  |  | **OR 1.14**  (1.12 to 1.16) | **0 fewer per 1,000**  (from 0 fewer to 0 fewer) | ⊕⊕OO  Low | CRITICAL |
| **association between the dementia and TyG index (analyzed as a continuous variable)** | | | | | | | | | | | | |
| 2 | observational studies | serious^a^ | not serious | not serious | not serious | strong association  all plausible residual confounding would reduce the demonstrated effect |  |  | **OR 1.37**  (1.03 to 1.83) | **0 fewer per 1,000**  (from 0 fewer to 0 fewer) | ⊕⊕⊕O  Moderate | CRITICAL |

CI: confidence interval; MD: mean difference; OR: odds ratio; TyG index: triglyceride-glucose index

**Explanations**

a. not analysis of publication bias

b. I2=85%

c. I2=82%

**Supplementary Figure 1. Sensitivity analysis of the association between triglyceride-glucose index and the risk of cognitive impairment** (A:category analysis; B: continuity analysis) **and dementia** (C:category analysis; D: continuity analysis).

**
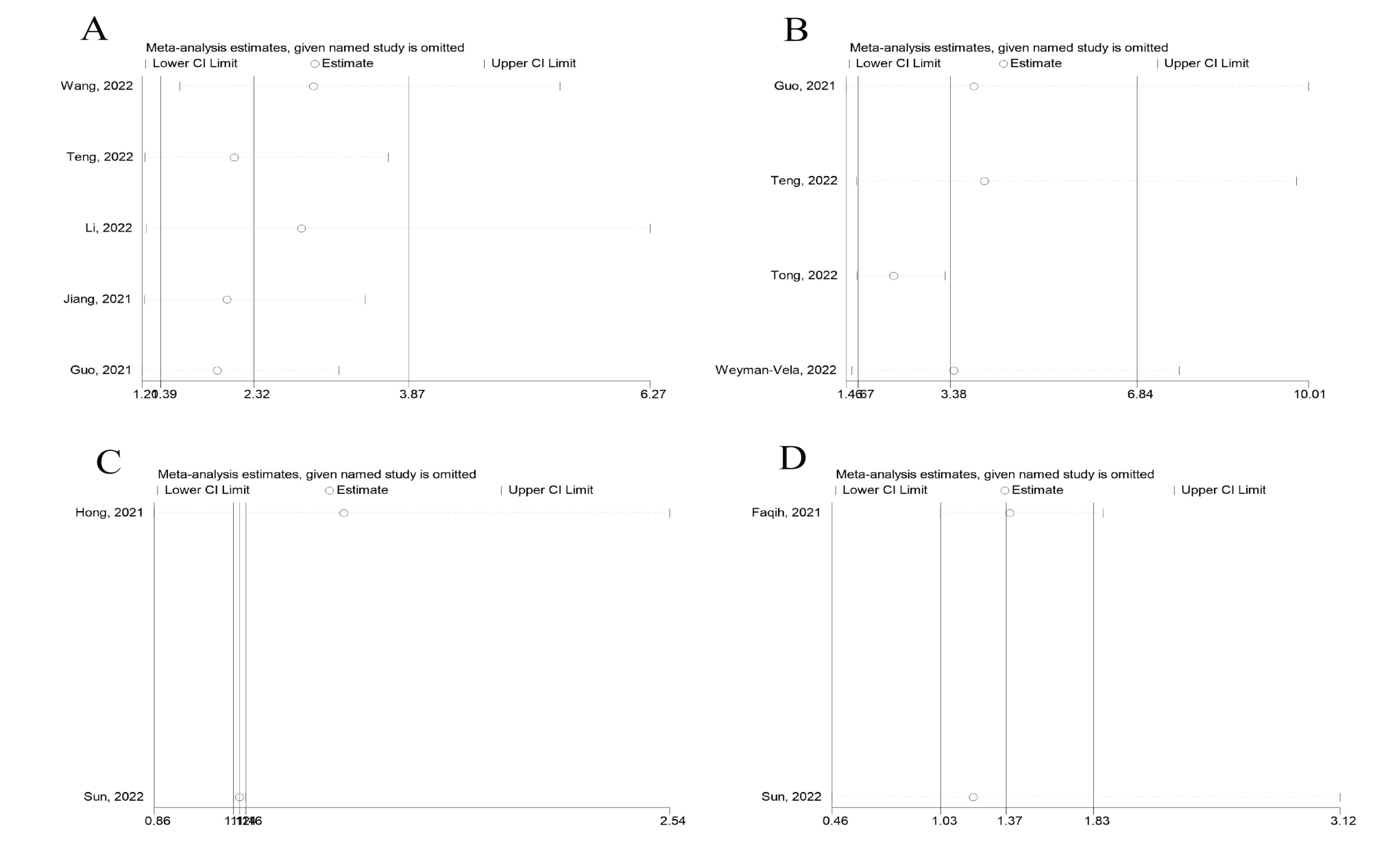
**
